# The degree of agreement between score-based decision and clinician's discretion regarding the need for tracheotomy in oral cancer surgery: A retrospective analysis

**DOI:** 10.1097/MD.0000000000026712

**Published:** 2021-07-30

**Authors:** Atsushi Abe, Yu Ito, Hiroki Hayashi, Hiroshi Furuta, Takanori Ishihama, Moriyasu Adachi

**Affiliations:** a Department of Oral and Maxillofacial Surgery, Nagoya Ekisaikai Hospital, Nagoya, Japan; b Department of Oral and Maxillofacial Surgery, Shizuoka General Hospital, Shizuoka, Japan.

**Keywords:** κ coefficient, airway management, complication, tracheotomy

## Abstract

In oral cancer surgery, the decision to perform a tracheotomy is often determined by the surgeon. In this study, we investigated the competency of clinical scoring systems in identifying patients who require tracheotomy and examined the degree of agreement between the surgeon's decision and the indications of various scoring systems. We identified 110 patients who were surgically treated for oral cancer. Of these, 67 patients (44 men and 23 women) who underwent resection and reconstruction were retrospectively analyzed. To derive the score, we evaluated the endpoint of the airway management score using clinical records and images. We divided the patients into two groups based on the Cameron and Gupta scores (tracheotomy and no-tracheotomy groups) and evaluated the degree of agreement with the surgeon's decision by calculating the κ coefficient. The κ coefficients of the Gupta and Cameron scores were 0.61 (95% confidence interval [CI]: 0.40–0.82) and 0.60 (95% CI: 0.38–0.82), respectively. The clinical evaluation of the κ coefficient indicated that the Cameron and Gupta scores agreed fairly with the surgeon's decision. In this study, the Cameron and Gupta scores fairly agreed with the decision of experienced surgeons and were confirmed as acceptable guides for making clinical judgments.

## 1 Introduction

Surgical treatment of oral cancer varies greatly depending on individual cases, some of which may require local excision or reconstruction. Such surgical invasions pose a risk of various postoperative complications, including surgical wound infection, necrosis of reconstructed flaps, postoperative bleeding, and upper airway obstruction. Postoperative complications may prohibit healing, extend the length of hospital stay, and be life-threatening.^[1–5]^ notably, the causes of airway obstruction include postoperative hematoma, pharyngolaryngeal edema, and morphological changes of the airway; thus, appropriate airway management is required.^[6,7]^ There are three methods of postoperative airway management:

1. extubation,
2. endotracheal intubation under sedation, and
3. tracheostomy.

Currently, tracheostomy or prolonged intubation remains the major modality of airway management for patients with oral and oropharyngeal cancers undergoing major surgery. However, no clear criteria currently exist for determining which method to select; therefore, the method is often determined on the basis of the surgeon's experience, considering interinstitutional differences and patient characteristics. Tracheostomy has been reported to be a reliable form of airway management; however, complications occur in 8–45% of cases.^[8]^ Several studies have been conducted to identify appropriate airway management strategies for the postoperative management of oral cancer.^[9–12]^

These methods may not be applicable for all cases because of disparities between institutions or differences in patient backgrounds. Enforcement of tracheotomy as an endpoint was determined by a physician. Therefore, it is necessary to examine the agreement between interrater evaluations of the need for tracheotomy and to further standardize the evaluation process. However, such examinations were not performed. In clinical practice, there is no confusion in judging cases in which tracheostomy is clearly necessary or clearly unnecessary. However, it is important to identify cases in which the need for tracheostomy is less obvious and difficult to judge, and can potentially lead to serious incidents.

The purpose of this study was to calculate the kappa coefficient (κ) to examine the degree of agreement between the physician's subjective evaluation and tracheotomy score evaluations.

## 2 Materials and methods

We performed a retrospective analysis of all patients who underwent resection and primary flap reconstruction in our department. A total of 110 patients with oral cancer (76 men and 34 women) who were treated with surgical methods under general anesthesia at the Department of Oral and Maxillofacial Surgery, Nagoya Ekisaikai Hospital, Nagoya, Japan, between January 2007 and April 2018. Forty-three patients who only received local excision and cerclage were excluded from the analysis because the risk of postoperative airway obstruction in such cases was expected to be low. The remaining 67 patients (44 men, 23 women) were included in the study. The study subjects were patients who underwent either broad resection of the primary lesion, followed by epidermization or major composite resection with reconstruction. Cancer staging was performed on the basis of inspection, contrast-enhanced computed tomography, magnetic resonance imaging, and positron emission tomography–computed tomography. Surgical procedures and methods for airway management for all patients were discussed and selected during a tumor conference in our department. Airway management methods were determined based on their experience by physicians who were found to be unaware of the tracheostomy score. The decision for postoperative airway management was based on the operator's experience. Usually, large tumors (stage T4) in the mouth floor or posterior lesions on the tongue and bilateral neck dissection are considered for elective tracheotomy. Various factors contribute to the need for tracheotomy in patients, ranging from

1. the extent of surgical resection to patient background,
2. resection procedure, inclusion/exclusion of neck dissection, and reconstruction procedure;
3. method of airway management; and
4. scoring based on previously reported indices.^[8–11]^ The parameters of the indices were evaluated and scored on the basis of patients’ medical and imaging records.

Patients evaluated using the Cameron and Gupta scores were divided into two groups: those requiring tracheotomy (tracheotomy group) and those not requiring tracheostomy (no-tracheotomy group). Agreement with the actual performance or non-performance of tracheotomy was evaluated using the κ coefficient. The physicians who participated in the conference were not informed of the study. Scores were also assessed by physicians who did not participate in the conference. Evaluation items that make up the score were studied to determine their influence on the need for tracheotomy in a patient. A tracheotomy score, which was adopted from the scoring system recommended by Cameron et al^[10]^ was used to evaluate the state of the patient's airway based on the type of operation. The agreement between the surgeon's decision to perform a tracheostomy and the evaluation of the tracheostomy score was analyzed using kappa statistics.

### 2.1 Tracheostomy scoring

Assessment of the indication for tracheostomy was based on that reported by Cameron^[10]^ and Gupta.^[12]^

Cameron's score^[10]^ evaluates the factors that influence the decision to perform elective tracheostomy in head and neck malignancy surgery. The score is divided into four main categories: tumor site, mandibular resection, neck dissection, and reconstruction. Each domain was assigned a score according to the most clinically important factor. If the sum of these four scores was ≥5, the risk of upper airway obstruction was high, and elective tracheostomy was considered.

Gupta's score^[12]^ assesses a priori predictors and the need for perioperative tracheostomy. Ten factors were established to predict the need for perioperative tracheostomy. These factors were divided into major and minor categories. Two points were assigned for each major risk factor and one point for each minor risk factor. Gupta's score was the sum of the scores for the major and minor risk factors. A score of 0–6 indicates no need for a tracheostomy, whereas a score of ≥7 indicates the need for a tracheostomy. The physician who assessed the score was different from the physician who decided on the tracheostomy and surgical technique. In addition, the physician who decided on the tracheostomy and procedure was unaware of the existence of the score.

### 2.2 Patient anonymity and informed consent

The present retrospective cohort study was approved by the Nagoya Ekisaikai Hospital Institutional Review Board (approval number: 2018–009), and written informed consent was obtained from all patients. All procedures were performed in accordance with the ethical standards of the International and/or National Research Committee and in accordance with the 1964 Declaration of Helsinki. The study was conducted in accordance with the Strengthening the Reporting of Observational Studies in Epidemiology (STROBE) statement: guidelines for reporting observational studies.

### 2.3 Statistical analysis

The κ coefficient was used to evaluate reliability among evaluators and to compare the different methods with regard to the number of scores identified. The κ coefficient was used instead of the intraclass correlation coefficient for ordinal scale scores. The agreement between the surgeon's decision and airway management suggested by the scores was analyzed using the κ coefficient. Statistical significance was set at *P* < .05. All statistical analyses were performed using EZR software (Saitama Medical Center, Jichi Medical University, Saitama, Japan), which is a graphical user interface for R (R Foundation for Statistical Computing, Vienna, Austria). More precisely, it is a modified version of R Commander designed to add statistical functions frequently used in biostatistics.^[13]^

## 3 Results

Patient characteristics, including tumor site, operative approach, and postoperative airway management, are shown in Table 1. Patients were aged between 42 and 88 years (mean age, 63.4 ± 11.0 years). Primary lesion excision was performed in 24 patients, extubation was performed in 21 patients, intubation under sedation was performed in 1 patient, and tracheotomy was performed in 2 patients. Primary lesion excision and neck dissection were performed in 30 patients, extubation was performed in 1 patient, intubation under sedation was performed in 8 patients, and tracheotomy was performed in 21. As a result of metastasis, neck dissection was performed in only 13 patients, and extubation was performed in all patients (Table 2).

**Table 1:** Patient characteristics.

		Airway management			
Factor	Group / Score	Immediate extubation	Overnight intubation	Tracheostomy	*P* value
n		35	9	23	
Age		63.5 ± 11.6	64.1 ± 9.3	63.0 ± 11.5	.968
Sex	Male	26 (38.8)	2 (3.1)	16 (23.9)	.012
	Female	9 (13.4)	7 (10.4)	7 (10.4)	
Stage	1	7 (10.4)	0 (0.0)	0 (0.0)	<.001
	2	15 (22.4)	0 (0.0)	0 (0.0)	
	3	7 (10.4)	7 (10.4)	15 (22.4)	
	4	2 (3.0)	2 (3.0)	8 (12.0)	
	Delayed cervical lymph node metastasis	4 (6.0)	0 (0.0)	0 (0.0)	
Tumor site	Maxilla	4 (6.0)	1 (1.5)	0 (0.0)	.841
	Buccal	4 (6.0)	2 (3.0)	3 (4.5)	
	Mandible	6 (9.0)	2 (3.0)	5 (7.5)	
	Floor of mouth	3 (4.5)	1 (1.5)	2 (3.0)	
	Tongue	18 (26.6)	3 (4.5)	13 (19.4)	
Cameron score	0	16 (23.8)	1 (1.5)	0 (0.0)	<.001
	1	14 (20.8)	3 (4.5)	5 (7.5)	
	2	5 (7.5)	2 (3.0)	3 (4.5)	
	3	0 (0.0)	0 (0.0)	1 (1.5)	
	4	0 (0.0)	1 (1.5)	0 (0.0)	
	5	0 (0.0)	1 (1.5)	8 (11.9)	
	6	0 (0.0)	0 (0.0)	5 (7.5)	
	7	0 (0.0)	1 (1.5)	1 (1.5)	
Gupta score	0	5 (7.5)	0 (0.0)	1 (1.5)	<.001
	1	19 (28.4)	0 (0.0)	0 (0.0)	
	2	10 (14.9)	3 (4.5)	1 (1.5)	
	3	1 (1.5)	1 (1.5)	2 (3.0)	
	4	0 (0.0)	1 (1.5)	2 (3.0)	
	5	0 (0.0)	3 (4.5)	4 (6.0)	
	6	0 (0.0)	0 (0.0)	4 (6.0)	
	7	0 (0.0)	0 (0.0)	1 (1.5)	
	8	0 (0.0)	1 (1.5)	2 (3.0)	
	9	0 (0.0)	0 (0.0)	3 (4.5)	
	10	0 (0.0)	0 (0.0)	1 (1.5)	
	11	0 (0.0)	0 (0.0)	2 (3.0)	

**Table 2:** Details of surgical data (only primary lesion excision, primary lesion excision and neck dissection and only neck dissection.

		Extubation	Intubation under sedation	Tracheotomy	Total
	Only primary lesion excision				
Primary lesion excision only	Segmental mandibulectomy	1			
	Marginal mandibulectomy	2			
	Resection of oral floor	3			
	Partial maxillectomy	2	1		
	Partial glossectomy	12		2	
	Resection of buccal mucosa	1			
	Subtotal	21	1	2	24
	Primary lesion excision and neck dissection				
Primary lesion excision and neck dissection	Segmental mandibulectomy		1	4	
	Marginal mandibulectomy		1	1	
	Resection of oral floor		1	2	
	Subtotal glossectomy			4	
	Hemiglossectomy			5	
	Partial glossectomy		3	3	
	Resection of buccal mucosa	1	2	2	
	Subtotal	1	8	21	30
Neck dissection only	Only neck dissection	13			13
Total	Total	35	9	23	67

**Figure 1:**
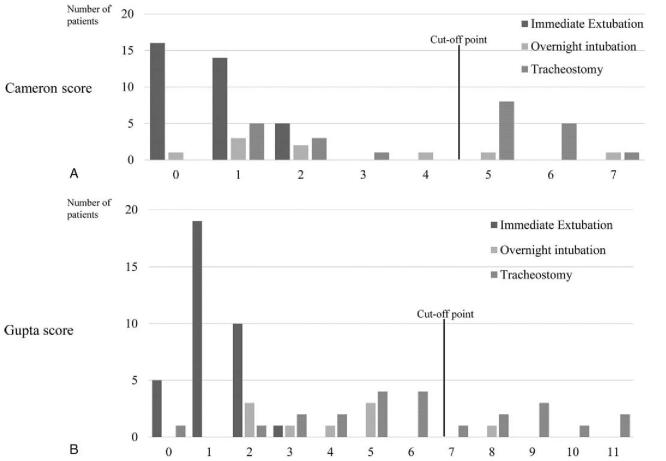
Number of patients in the airway management group, according to the tracheostomy score. (A) Cameron score. (B) Gupta score. The threshold for the Cameron score was 5. Patients at or above the threshold are at an increased risk of upper airway compromise. The threshold for the Gupta score is 7. Total score ≤6: Suggestive of no need for a tracheostomy and Total score ≥7: Indicative of a need for a tracheostomy.

Detailed results of scores using the methods reported by Cameron et al^[10]^ and Gupta et al^[12]^ are shown in Figure 1 (A and B) and Table 3 (A and B). Scoring using the methods reported by Cameron and Gupta clearly indicated whether a patient required a tracheotomy. The number of patients who suggested requiring tracheotomy was within 16 patients according to the Cameron score and 10 patients by Gupta score. Tracheotomy was performed in 9 patients in the no-tracheotomy group, as rated by the Cameron score (false negative). The details of these 9 patients were as follows: partial glossectomy and total neck dissection (n = 3), posterior partial glossectomy (n = 2), buccal mucosa and total neck dissection (n = 2), marginal mandibulectomy (n = 1), and segmental mandibulectomy (n = 1). Tracheotomy was performed in 14 patients in the no-tracheotomy group, on the basis of Gupta's score (false negative). The details of the patients undergoing tracheotomy in the no-tracheotomy group rated by the Gupta score are as follows: partial glossectomy and total neck dissection (n = 3), posterior partial glossectomy (n = 2), hemiglossectomy, forearm flap reconstruction, and total neck dissection (n = 4); subtotal glossectomy, forearm flap reconstruction, and total neck dissection (n = 3), marginal mandibulectomy plate reconstruction (n = 1) and total neck dissection and oral floor resection (n = 1).

**Table 3:** Comparison of clinical scores.

A				
		Surgeons’ decision traceostomy	No tracheostomy	
Cameron score	Traceostomy need	16	2	18
	No tracheostomy	9	42	51
		25	44	69

		Surgeons’ decision		
		Traceostomy	No tracheostomy	
Gupta score	Traceostomy need	9	1	10
	No tracheostomy	14	43	57
		23	44	67

B		
	Cameron	Gupta
Suggested tracheostomy	16	10
True positive	14	9
False positive	2	1
False negative	9	14
True negative	42	43
Sensitivity	0.61	0.39
Specificity	0.96	0.98
Positive predictive value	0.88	0.9
Negative predictive value	0.82	0.75
Likelihood ratio for positive results	13.39	17.22
Likelihood ratio for negative results	0.41	0.62

Tracheotomy was not performed in 2 patients in the tracheotomy group, as rated by the Cameron score (false positive). One was sedated and intubated for total neck dissection, buccal mucosal resection, and mandibular segmental resection, and the other was sedated and intubated for mandibular segmental resection, hard tissue reconstruction, and total neck dissection. One patient in the tracheotomy group, as rated by the Gupta score, did not undergo tracheostomy. The patient was intubated for hard tissue reconstruction, total neck dissection, and segmental mandibulectomy (Fig. 1 A and B).

Regarding the accuracy of each score, sensitivity was within the range of 0.64 (Cameron Score) and 0.39 (Gupta Score) and specificity was within the range of 0.96 (Cameron Score) and 0.98 (Gupta Score). The sensitivity was low, and specificity was high for all scores. The positive predictive value (PPV) range was between 0.88 (negative Score) and 0.9 (Gupta Score), and negative predictive value (NPV) ranged between 0.82–0.75. The positive likelihood ratio was within the range of 13.39 and 17.22, and the negative likelihood ratio was within the range of 0.41–0.62. These diagnostic tests, with low sensitivity and high specificity, are good for including patients with positive results; in the current case, those patients predicted to undergo tracheostomy should undergo surgery. This was supported by the high positive likelihood ratio (13.9, 17.22) and the intermediate negative likelihood ratio (0.41, 0.62). The κ coefficients of the Gupta and Cameron scores were 0.61 (95% CI, 0.4–0.82) and 0.6 (95% CI, 0.38–0.82) (Tables 3 and 4).

**Table 4:** Agreement (kappa) between the clinical scores (the relations between each score and surgeons’ decisions).

Score	κ coefficient	95% CI
Gupta	0.61	0.40–0.82
Cameron	0.6	0.38–0.82

## 4 Discussion

Patients at risk of airway obstruction during oral cancer surgery should undergo elective tracheostomy, however, complications such as bleeding, occlusion, local infection, and pneumonia occur at a rate of 4–8% in tracheotomy.^[16–20]^ As these complications prolong the patient's recovery and lengthen the hospital stay, the appropriate strategy for airway management is controversial. In many cases, elective tracheostomy is determined on the basis of the surgeon's experience, which leads to variability. It is not difficult to determine which cases require tracheostomy. However, cases involving resection of the posterior tongue, mandible, and floor of the mouth can make it difficult to decide whether to perform a tracheostomy. Such cases are at high risk of upper airway obstruction, which can lead to serious incidents, making it necessary to identify such cases. Research is needed to establish the criteria for tracheostomy indications to ensure appropriate airway management. This study examined the degree of agreement between physician ratings and tracheostomy scores regarding the need for tracheostomy. After the assessment, the κ coefficients of the Gupta and Cameron scores were 0.61 (95% CI: 0.4–0.82) and 0.6 (95% CI: 0.38–0.82), respectively. Moderate congruity was found between the physician's evaluation and tracheotomy scores’ evaluations. In this study, the Cameron and Gupta scores agreed with the surgeon's judgment to some extent, and they were confirmed to be able to be adapted to clinical judgment in the hospital setting. These values are affected by the prevalence; however, the scores are effective for screening postoperative airway management.

Airway obstruction after oral cancer surgery occurs as a result of a combination of multiple factors, such as large-area excision of the mandible, tongue, and floor of the oral cavity, bilateral neck dissection, use of bulky reconstruction flaps, postoperative hematoma and pharyngolaryngeal edema, and relaxation of the tongue muscle.^[14,15]^ To avoid critical situations occurring as a result of postoperative airway obstructions, pathophysiological observations, such as those based on tracheal tug, neck ultrasound, oxygen saturation, and monitoring by a capnometer, are necessary. Although these methods are essential for monitoring respiratory management, they are not reliable. In patients with difficult postoperative airway management, emergency tracheostomy is required because of upper airway obstruction. There are several reports on assessment methods to predict the need for elective tracheostomy in patients with oral cancer to avoid such serious situations.^[9–12]^ However, these studies may not be applicable to all patients because of disparities between institutions and differences in patient backgrounds; therefore, we investigated cases in which tracheostomy was necessary on the basis of these evaluation methods and the surgeon's decision diverged.

Cameron et al^[10]^ also reported a method that can be used to identify patients who require tracheotomy. This study evaluated the location of the tumor, extent of mandibular resection, method of neck resection, and method of reconstruction and examined the relationship between surgical technique and airway management. This scoring system was created by combining elements used in the evaluation on the basis of the same items. Benatar–Haserfaty et al^[23]^ analyzed the application of elective tracheostomy with a cutoff Cameron score of ≥5. The results showed a diagnostic sensitivity value of 0.7 (95% confidence interval [CI] 0.57–0.82), diagnostic specificity value of 0.9 (95% CI 0.79–0.99), PPV of 0.9 (95% CI 0.81–0.99), and NPV of 0.67 (95% CI 0.54–0.8). The Cameron score, based on objective data, can enhance the decision to perform elective tracheostomy for oral tumor surgery.

Gupta et al^[12]^ reported scoring based on small and large categories, including resection, reconstruction methods, previous experience of radiotherapy, and degree of mouth opening, which may obstruct airway management even before surgery. Gupta et al^[12]^ reported that the sensitivity of the clinical assessment scoring system for tracheotomy (CASST) was 95.5%, selectivity was 99.5%, positive predictive value (PPV) was 96.9%, and negative predictive value (NPV) was 99.3%. Sensitivity was low in our patients, although the selectivity and PPV were greater than 90%.

In the present study, the Cameron score had a sensitivity of 0.64 and specificity of 0.96. The sensitivity of the Gupta score was 0.39, and the specificity was 0.98. This result showed low sensitivity and high specificity, similar to findings from previous studies. In this system, scores are given for surgery (especially areas of resection) and reconstruction procedures. In terms of prediction of airway management associated with surgery, details of surgery are incorporated within this system in comparison with the other systems; therefore, this system is expected to be useful for surgeons. Whether high sensitivity or high selectivity is required for these tests depends on the clinical state and study population. Because a number of analyses showed false-negative results, depending on the criteria, scores became relatively low in partial glossectomy, as well as in cases in which forearm flap reconstruction was performed with pull-through or supraomohyoid neck dissection. Whether to perform tracheotomy or to simply maintain intratracheal intubation under sedation in such cases is controversial. In addition to a system that can be used to distinguish at-risk patients from those with false-negative results, it is important to combine several systems for evaluation. Similarly, false-positive diagnoses for tracheotomy must be avoided in patients who do not require this procedure. The reason the patients who needed tracheotomy on the basis of these scores actually did not undergo tracheostomy was considered as follows. The Cameron and Gupta scores were high in patients who underwent resection of the mandibular area and surrounding tissue (e.g., buccal mucosa or floor of mouth) and hard tissue reconstruction. However, in these cases, the surgeons decided that intubation under sedation was possible when postoperative aspiration was not a concern. We confirmed that the Cameron and Gupta scores were consistent with the surgeon's decision to some extent and could be applied generally to clinical decisions. The results showed that the two scores had similar sensitivity and specificity, but Gupta's score was more useful for assessing the nature of the surgical procedure. Many cases in which there was a discrepancy between the score assessment and tracheostomy were associated with resection of the suprahyoid muscle group. It was assumed that decisions were divided in such cases.

These scorings were based on weighted evaluation criteria, such as tumor location, neck dissection type, systemic disease, and reconstruction procedure. Specificity, PPV, NPV, and positive/negative likelihood ratios were generally high in this study. These values are affected by the prevalence, although scores are effective for screening postoperative airway management. Schmutz et al^[21]^ reported that patient populations differ by institution; therefore, they failed to predict the need for tracheotomy on the basis of these clinical scoring systems. Similarly, Lee et al^[22]^ reported that they could not identify correlations between the need for tracheotomy and the clinical findings in patients with oral cancer based on the Cameron score. Moreover, Benatar–Haserfaty et al^[23]^ conducted an analysis based on the Cameron score for performing elective tracheotomy in oral cancer surgery. The analysis revealed high selectivity and PPV (90% for both), low sensitivity (70%), and NPV (67%), making it difficult to determine whether tracheotomy was necessary. Upon reviewing reports that reevaluate these scores, patients who actually require tracheotomy may not be accurately identified, and tracheotomy is suggested in a large proportion of cases. This is likely due to large differences in the choices of surgical methods, decisions based on surgeons’ experience, and patient population. Moreover, postoperative hematoma and pharyngolaryngeal edema cannot be predicted directly from the scores investigated in this study. Clinically, in such cases, airway obstruction rapidly advances and becomes irreversible. Intratracheal intubation or tracheotomy must be selected during emergencies; these urgent decisions are the largest problem. These scoring systems were reported years ago, and since then, there have been advancements in equipment (e.g., energy devices), improvements in surgical techniques and perioperative care, and extensive changes in the applicability criteria of surgical procedures. The establishment of a scoring system that accommodates such advancements and changes is required.

This study has a few limitations. Notably, we excluded patients who underwent cerclage because the risk of postoperative airway obstruction was expected to be low in such cases. However, there have been reported cases of severe outcomes resulting from occlusions, even if resection was performed only on the frontal part of the mandible, as well as in cases in which only the primary lesion was excised or a single neck dissection was performed.^[22,23]^ Even in cases that appear to be low risk, resection or abrasion of the genioglossus muscle, geniohyoid muscle, or mylohyoid muscle may cause deterioration of airway obstruction due to the loss of support of the hyoid bone. Such procedures with moderate surgical invasion are managed outside the intensive care unit and therefore pose a risk of delayed treatment of airway obstruction. The evaluation of such cases will be required in the future.

Importantly, postoperative hematoma and pharyngolaryngeal edema cannot be predicted directly from the scores investigated in this study; therefore, scoring systems that can accommodate such changes should be established.

## Acknowledgments

We would like to thank Editage (www.editage.com) for their writing support.

## Author contributions

**Conceptualization:** Atsushi Abe.

**Data curation:** Hiroki Hayashi.

**Formal analysis:** Takanori Ishihama.

**Investigation:** Moriyasu Adachi.

**Methodology:** Yu Ito.

**Writing – review & editing:** Hiroshi Furuta.
